# Plant-Made Nervous Necrosis Virus-Like Particles Protect Fish Against Disease

**DOI:** 10.3389/fpls.2019.00880

**Published:** 2019-07-09

**Authors:** Johanna Marsian, Daniel L. Hurdiss, Neil A. Ranson, Anneli Ritala, Richard Paley, Irene Cano, George P. Lomonossoff

**Affiliations:** ^1^John Innes Centre, Norwich Research Park, Norwich, United Kingdom; ^2^Astbury Centre for Structural Molecular Biology, School of Molecular and Cellular Biology, University of Leeds, Leeds, United Kingdom; ^3^VTT Technical Research Centre of Finland, Espoo, Finland; ^4^Centre for Environment Fisheries and Aquaculture Science (Cefas), Weymouth Laboratory, Weymouth, United Kingdom

**Keywords:** Atlantic cod nervous necrosis virus, virus-like particles, transient expression, sea bass, protective immunity

## Abstract

Virus-like particles (VLPs) of the fish virus, Atlantic Cod Nervous necrosis virus (ACNNV), were successfully produced by transient expression of the coat protein in *Nicotiana benthamiana* plants. VLPs could also be produced in transgenic tobacco BY-2 cells. The protein extracted from plants self-assembled into *T* = 3 particles, that appeared to be morphologically similar to previously analyzed NNV VLPs when analyzed by high resolution cryo-electron microscopy. Administration of the plant-produced VLPs to sea bass (*Dicentrarchus labrax*) showed that they could protect the fish against subsequent virus challenge, indicating that plant-produced vaccines may have a substantial future role in aquaculture.

## Introduction

Nervous necrosis virus (NNV) is the causative agent of viral nervous necrosis (VNN) that is a major viral pathogen of fish known to affect over 40 cultured marine fish species worldwide (Tan et al., 2001; Walker and Winton, 2010). Nodavirus infection leads to extensive economic losses to the aquaculture industry each year (Lin et al., 2007). The virus replicates in cells of the brain, spinal cord, and retina causing lesions leading to viral encephalopathy and retinopathy (VER), followed by skin darkening, abnormal swimming behavior and massive mortality (Grotmol et al., 1997; Bovo et al., 1999; Thiery et al., 1999). VNN often causes mortality rates up to 100% in larvae and early juvenile stages and can result in rapid loss of the hatchery; adult fish are also susceptible, but mortality rates are lower (Lin et al., 2007). Thus, the development of a cost- effective and viable vaccine is of great interest to the aquaculture industry.

Nervous necrosis virus is a member of the family *Nodaviridae* that contains two genera: *Alphanodavirus*, which usually infects insects, and *Betanodavirus*, also known as piscine nodavirus (Mori et al., 1992). *Betanodavirus* is currently classified into four genotypes: striped jack NNV (SJNNV), tiger puffer NNV (TPNNV), barfin flounder NNV (BFNNV) and red-spotted grouper NNV (RGNNV) (Nishizawa et al., 1997) with a fifth, turbot NNV (TNNV), proposed (Johansen et al., 2004) and a further three unclassified viruses known (Sahul Hameed et al., 2019). The four genotypes fall into three serotypes with RGNNV and BFFNV sharing serotype C. Each of these genotypes infects a number of different fish species but all share similar features (World Organisation for Animal Health [OIE], 2018).

Virions of the *Nodaviridae*, including NNV, are non-enveloped, 25–30 nm in diameter and contain 180 copies of a single type of coat protein subunit arranged with *T* = 3 symmetry. The particles contain two molecules of positive-sense RNA, RNA1, and RNA2. RNA1 (3.1 kb) encodes the RNA-dependent RNA polymerase (102 kDa), critical for RNA transcription and replication, and the non-structural protein B (10 kDa), expressed from a subgenomic RNA (Friesen and Rueckert, 1982; Guarino et al., 1984; Saunders and Kaesberg, 1985) that is involved in inhibition of host RNA interference (Mori et al., 1992; Guo et al., 2003; Chao et al., 2005). RNA2 (1.4 kb) encodes the coat protein precursor alpha (43 kDa) and has an additional function in regulating the synthesis of RNA3 (Dasgupta et al., 1984; Friesen and Rueckert, 1984; Mori et al., 1992). The coat protein precursor self-assembles into *T* = 3 provirions (Friesen and Rueckert, 1981; Gallagher and Rueckert, 1988; Cheng et al., 1994). Provirions mature by spontaneous autocatalytic cleavage of the coat protein alpha post-assembly, producing proteins beta (38 kDa) and gamma (5 kDa) which remain part of the mature virion (Hosur et al., 1987).

Virus-like particles (VLPs), which resemble authentic virions but lack the infectious genome, have proven to be effective immunogens against a variety of diseases (Grgacic and Anderson, 2006; Bachmann and Jennings, 2010). Several potential vaccines against NNV based on VLPs have been investigated but none has been marketed yet. For example, Lin et al. (2001) used Sf21 insect cells and a recombinant baculovirus vector to express the capsid protein from Malabar grouper (*Epinephelus malabaricus*) NNV (MGNNV) and showed that the protein assembled into VLPs. These had a similar size and geometry to the native virus and, as well as the coat protein, were found to contain random host-derived RNA of different sizes (Lin et al., 2001). Using *Escherichia coli*-based expression, Lu et al. (2003) and Liu et al. (2006) produced VLPs for Dragon grouper (*Epinephelus lanceolatus*) nervous necrosis virus (DGNNV) and showed that the particles were able to block attachment of native virus to the surface of fish nerve cells in culture and to raise antibodies in vaccinated fish. Lai et al. (2014) also showed that *E. coli*-produced orange-spotted grouper nervous necrosis virus (GNNV) VLPs could stimulate the production of high titre antibodies in fish and Chen et al. (2015) determined the crystallographic structures of the *E. coli*-expressed VLPs to 3.6 Å resolution. Most recently, Xie et al. (2016) solved the structure of GNNV VLPs to 3.9 Å by cryo-electron microscopy and identified sites on the assembled capsids for insertion of foreign peptides; they also showed that the VLPs could elicit an antibody response in Asian sea bass. Moreover, *E. coli*-expressed VLPs have been shown to elicit a strong cellular and innate immune response in orange-spotted grouper, in particular the complement system, an important component of the innate immune system and anti-viral immunity in teleost (Lai et al., 2014).

In the last 20 years, plant-based expression systems have become serious competitors to bacteria, insect cells, yeast or mammalian cells as production systems for pharmaceutical materials (Lomonossoff and D’Aoust, 2016). They have the potential to produce vaccines cheaply, a major consideration if they are to be deployed in aquaculture. Plants also appear to be particularly suitable for the production of VLPs (Thuenemann et al., 2013a; Marsian and Lomonossoff, 2016), some of which have been shown to be capable of providing protective immunity (Thuenemann et al., 2013b; Marsian et al., 2017). We therefore, examined whether plant systems could be used to produce a candidate vaccine for deployment in aquaculture. Here, we show that both transient expression of the coat protein of Atlantic cod nervous necrosis virus (ACNNV) in *Nicotiana benthamiana* leaves or in transgenic tobacco BY-2 cells leads to the production of VLPs. Cryo-EM analysis of the particles produce in *N. benthamiana* leaves resulted a 3.7 Å resolution reconstruction, which confirmed ACNNV LPs have a similar structure to those previously reported for GNNV. When administered either intraperitoneally or intramuscularly to sea bass, the VLPs were shown to confer partial protection against subsequent challenge with ACNNV, indicating that plants can be used to produce effective vaccines for use in aquaculture.

## Materials and Methods

### Plasmid Constructs

The gene sequence for the ACNNV coat protein (GenBank Accession No. EF617326.1) was codon-optimized for *N. benthamiana* and ordered for synthesis from GeneArt (Life Technologies) with flanking AgeI and XhoI sites. The gene was cloned into an AgeI/XhoI- digested pEAQ-*HT* (Sainsbury et al., 2009) to produce pEAQ-*HT*-NNV. *Agrobacterium tumefaciens* LBA4404 were transformed with the construct by electroporation.

### Transient Expression in *N. benthamiana*

*Agrobacterium tumefaciens* containing pEAQ-*HT*-NNV was grown to stable phase at 28°C in Luria–Bertani medium supplemented with 50 μg/ml kanamycin and 50 μg/ml rifampicin. The culture was then pelleted by centrifugation at 2500 × *g* and re-suspended in MMA buffer (10 mM MES, pH 5.6, 10 mM MgCl_2_, 100 μM acetosyringone) to an OD_600_ of 0.4. The bacteria were left at room temperature for 0.5–3 h prior to infiltration. The suspensions were pressure infiltrated into the leaves of 3-week-old *N. benthamiana* plants as described by Thuenemann et al. (2013b).

### Extraction and Purification of ACNNV VLPs From Leaves

Infiltrated leaf tissue was weighed and homogenized using a Waring (Torrington, CT) blender with 3× volume of extraction buffer (0.1 M sodium phosphate, pH 7.0) plus added protease inhibitor according to the manufacturer’s instructions (Roche, Welwyn Garden City, United Kingdom) and then filtered through Miracloth (Calbiochem). The crude extract was centrifuged at 9,500 × *g* for 15 min at 4°C and the supernatant was then centrifuged through a sucrose cushion (1 ml 70% (w/v) and 5 ml 25% (w/v)) at 167,000 × *g* for 2.5 h at 4°C and the lower fraction retrieved. Following removal of residual sucrose by dialysis, the sample was further purified by centrifugation through a sucrose gradient (20–60% (w/v)) at 167,000 × *g* for 3 h at 4°C. VLPs were collected by piercing the bottom of the tube with a needle and retrieving each fraction. After dialysis to remove the sucrose, the VLPs were concentrated by pelleting in the TH641 ultracentrifuge swing-out rotor (Sorvall) for 1, 5 h at 197,819 × *g* at 4°C. The pellets were resuspended in a small volume of either PBS (140 mM NaCl, 15 mM KH_2_PO_4_, 80 mM Na_2_HPO_4_, 27 mM KCl, pH 7.2) or TBS (50 mM Tris-Cl, 0.3 M NaCl, 1 mM EDTA, pH 8.5).

### Stable Transformation of Tobacco BY-2 Cells

Two to three day-old BY-2 cell cultures were incubated with 0.25 mM acetosyringone and mixed with a suspension of *A. tumefaciens* carrying pEAQ-HT-NNV to an OD600 of 1.0. Both were mixed together and left to incubate at 28°C for 2–3 days. Afterwards the BY-2 cells were transferred onto solid Murashige and Skoog (MS) basal media with appropriate antibiotics (25 ppm kanamycin, 500 ppm carbenicillin and 500 ppm vancomycin) and left in the darkness at 28°C. Fifteen calli positive for the NNV coat protein gene were identified by PCR and further analyzed for protein expression by western blot analysis using an anti-NNV antibody (Abcam ab26812). 500 ml of MS media was inoculated with BY-2 cell line 16 and after 3.5 days the cells were harvested. Cells were homogenized using a mortar and pestle and the VLPs purified as described above.

### SDS-PAGE and Western Blot Analysis

Protein extracts were analyzed by electrophoresis on 4–12% (w/v) NuPAGE Bis-Tris gels (Life Technologies). Western Blot analyses were performed using a monoclonal primary antibody against the NNV coat protein (Abcam ab26812) followed by detection with a goat anti-rabbit secondary antibody conjugated to horseradish peroxidase and developed using the chemiluminescent substrate Immobilon Western (Millipore).

### Transmission Electron Microscopy

Virus-like particles were adsorbed onto plastic and carbon-coated copper grids, washed with several drops of water and then stained with 2% (w/v) uranyl acetate for 15–30 s. Grids were imaged using a FEI Tecnai G2 20 Twin TEM with a bottom-mounted digital camera.

### Cryo-EM Data Collection

Cryo-EM grids were prepared by placing 3 μl of 0.9 mg/ml ACNNV-LPs onto 200 mesh grids with 2-μm holes (Quantifoil R2/2, Quantifoil Micro Tools, GmbH, Germany). Grids were glow-discharged for 30 s prior to plunge-freezing in liquid ethane cooled by liquid nitrogen, using a Leica-EM GP at 85% relative humidity. Data was collected on a FEI Titan Krios (ebic, Oxford, United Kingdom) transmission electron microscope at 300 kV, with a total electron dose of ∼45e^–^ per Å^2^ and a final object sampling of 1.06Å per pixel. A total of 2359 exposures were recorded using the EPU automated acquisition software on a Gatan K2 Summit energy-filtered direct detector (Gatan, Inc.). Each exposure movie had a total exposure of 2 s and contained 20 images.

### Image Processing and 3D Reconstruction

Drift-corrected averages of each movie were created using MOTIONCOR2 (Zheng et al., 2017) and the contrast transfer function of each micrograph was determined using Gctf (Zhang, 2016). Automated particle picking was performed using Gautomatch^1^, before several rounds of reference-free 2D classification were carried out in the RELION2.0 pipeline (Scheres, 2012; Kimanius et al., 2016). After each round, the best classes were taken to the next step of classification. Icosahedral symmetry was imposed during 3D auto-refinement and post-processing was employed to appropriately mask the model, estimate and correct for the B-factor of the maps. The final resolutions were determined using the “gold standard” Fourier shell correlation criterion (FSC = 0.143). Local resolution was estimated in RELION2.0 which also generated a map filtered by local resolution. The ACNNV-LP cryo-EM map was deposited in the Electron Microscopy Data Bank under ID code EMD-4899.

### Atomic Model Building and Refinement

As a starting point for model building, a single asymmetric unit, comprising only the shell domain, of the GNNV VLP X-ray structure (PDB 4WIZ; Chen et al., 2015), was fitted into the ACNNV-LP EM density map using UCSF Chimera (Pettersen et al., 2004). In coot, the protein sequence was mutated to account for sequence differences between ACNNV and GNNV (Supplementary Figure S2) and fitted using the “real space refinement tool” (Emsley et al., 2010). The resulting model was then symmetrized in UCSF Chimera to generate the capsid and subject to refinement in Phenix (Headd et al., 2012). Iterative rounds of manual fitting in coot and refinement in phenix were carried out to improve non-ideal rotamers, bond angles and Ramachandran outliers (Supplementary Table S1). The coordinates for the ACNNV-LP asymmetric unit were deposited in the Protein Data Bank under the ID code PDB 6RJ0. Figures were generated using UCSF Chimera and PyMOL (The PyMOL Molecular Graphics System, Version 2.0 Schrödinger, LLC).

### Immunization of Fish and Subsequent Challenge

Unvaccinated sea bass (*Dicentrarchus labrax*) fry were obtained from a commercial hatchery (Gravelines, France) with no history of NNV and grown on to average size of 30.5 g (12.09 cm) in UV treated seawater. Fish were fed commercial pellet diet (Gemma Diamond 1.8, Skretting). Duplicate experimental tanks of 420 l volume each containing 140 fish were established and fish acclimated for 2 weeks before vaccination. Ten stock fish were terminated prior to the start of the study using an approved method and blood sampled then serum used to assess baseline immune status by enzyme linked immunosorbent assay (ELISA). Approximately 2 ml of purified plant-expressed ACNNV VLPs diluted in phosphate-buffered saline (PBS) were used for immunization. Protein concentration was determined by UV spectrophotometry at 280 nm (Nanodrop) and bicinchoninic acid (BCA) assay (Pierce) as per manufacturer’s instructions.

Fish were starved for 24 h prior to vaccination, anesthetized with 0.01% Tricaine Methane sulphonate (MS222) then injected with 50 μl of VLP diluted in PBS (5 μg/fish) or 50 μl PBS (controls). 35 fish were vaccinated by intramuscular (IM) injection with VLPs, 35 fish by intraperitoneal (IP) injection with VLPs, 35 fish IM injected with diluent (PBS) and 35 fish IP injected with diluent (PBS) in each tank. Fish were held at 25°C for 4 weeks (700° days) to allow development of any potential immune response. At this time, five fish from each group in each tank were terminated using an approved method and blood samples taken through the caudal vein. Sera were used to assess development of specific immune response by ELISA to detect anti-betanodavirus antibodies in serum. The ELISA test was performed as previously described by Taylor et al. (2010) modified with gradient purified RGNNV 378/102 as coating antigen and the mouse anti-European sea bass IgM monoclonal antibody (Aquatic Diagnostics, Stirling, United Kingdom).

Betanodavirus isolate 378/102 (RGNNV genotype) was propagated in the SSN-1 cell line (ECACC, 96082808) in Liebovitz’s L15 media supplemented with 2 mM glutamine, 10% fetal bovine serum (FBS), 100 units penicillin/ml and 100 μg streptomycin/ml with incubation at 25°C. Clarified cell culture supernatant containing virus was harvested and the titre determined by tissue culture infectious dose limiting dilution in SSN-1 cells. Virus harvest was diluted to 5 × 10^5^ TCID_50_/ml for injection challenge. At 35 days post-vaccination (875° days) fish were challenged by IM injection of 0.1 ml of cell culture supernatant containing betanodavirus (isolate 378/102) at 5 × 10^5^ TCID_50_/ml and held for a further 4 weeks of observation (Supplementary Figure S4). Fish showing clinical signs consistent with VER caused by nodavirus infection (darkening, separation from shoal, inability to maintain station, corkscrew swimming) were removed and terminated by an approved method when moribund. Whole brain and blood samples were taken from moribund terminated fish for subsequent serum analysis and confirmation of specific mortality (presence of nodavirus). Brain samples were collected throughout and stored at −80°C until analysis. Blood samples were allowed to clot overnight at 4°C serum removed and stored at −20°C until analysis. For virus isolation and confirmation of specific mortality, whole brains were thawed, homogenized in 10 volumes (w/v) cell culture medium in microfuge tubes with glass beads using a FastPrep 24 benchtop homogeniser (MPBio). Homogenates were clarified by centrifugation at 3000 *g* for 5 min and supernatants inoculated onto SSN-1 cells at 1:100 and 1:1000 dilution in 24 well cell culture plates. Cultures were incubated at 25°C for 7 days and observed for cytopathic effect by phase contrast light microscopy.

Survival probability of the different vaccinated and mock vaccinated groups during experimental challenge was represented using a Kaplan–Meier survival plot with 95% confidence intervals. Survival distribution was compared with a log-rank test to determine if statistical differences existed between the groups. A pairwise log-rank comparison was then performed between groups. Survival analysis was performed on replicate and then combined tank data per group.

## Results

### Expression of NNV VLPs in Whole Plants

**FIGURE 1:**
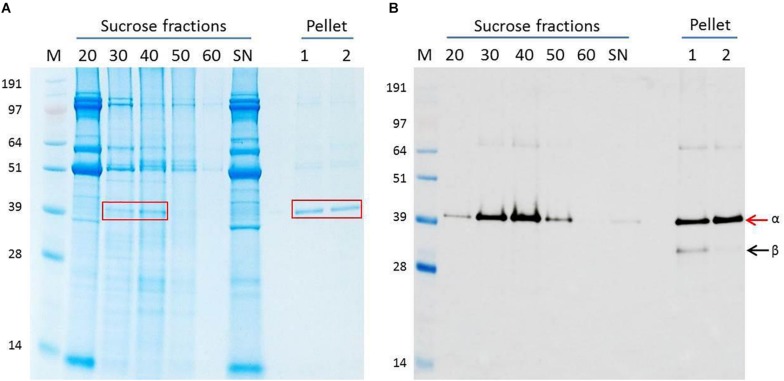
Purification of NNV VLPs. Clarified plant extract containing ACNNV VLPs was run through a sucrose gradient followed by pelleting. **(A)** Instant blue-stained SDS-PAGE gel and **(B)** western blot probed with anti-NNV (ab26812) antibody of the various stages of purification. The % sucrose in each fraction is indicated. S/N represents the sample before application to the sucrose gradient. The 30 and 40% sucrose fractions harboring the NNV VLPs were combined, dialyzed and then pelleted. Two different buffers were used for resuspension. Lane 1 = PBS buffer. Lane 2 = TBS buffer. *M* = SeeBlue Plus 2 with molecular weights indicated. Arrows indicate ACNNV coat protein alpha (α) or mature protein beta (β) in the western blot shown in **(B)** and the red boxes indicate the position of the ACNN.

The leaves of *N. benthamiana* plants infiltrated with pEAQ-*HT*-NNV were harvested 6 days post-infiltration (dpi) by which time they started to show slight signs of chlorosis. After homogenization of the tissue and initial purification, the plant extract was centrifuged through a 20–60% (w/v) sucrose step gradient and fractions analyzed by SDS-PAGE and western blot analysis. This revealed that the majority of the protein with the expected size of the uncleaved alpha peptide (43 kDa) occurred in the 30 and 40% sucrose fractions (Figure 1A) and that this material specifically reacted with anti-ACNNV antibodies (Figure 1B). The stained gel also revealed that these fractions contained significant amounts of additional host-derived proteins.

**FIGURE 2:**
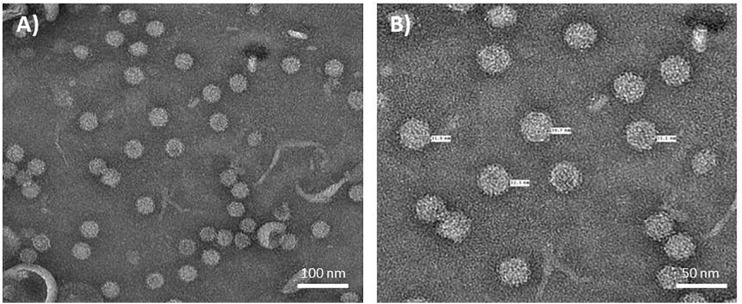
Electron micrographs of NNV VLPs. Plant extract containing ACNNV VLPs was purified through two sucrose gradients and concentrated by pelleting. ACNNV VLPs were visualized by negative staining with 2% (w/v) uranyl acetate and transmission electron microscopy. **(A,B)** represent two different magnifications with the diameters of selected particles shown in **(B)**.

To further purify the putative NNV VLPs, the 30 and 40% sucrose fractions were combined, dialysed and any VLPs pelleted by ultracentrifugation. The resulting pellets were resuspended in either PBS buffer or TBS buffer to examine the stability of the VLPs under different conditions. SDS-PAGE and western blot analysis suggested that both samples were very pure with few, if any contaminating proteins present (Figure 1A). A slight difference in the samples resuspended in the different buffers could be observed in the western blot B) where an additional lower molecular mass band could be seen in sample resuspended in PBS (lane 1). Most likely this protein, which was detected by the anti-NNV antibody, is the processed, beta, form of NNV coat protein (Hosur et al., 1987; Schneemann et al., 1992). As this band is not seen in the samples resuspended in TBS (Figure 1B, lane 2), it is likely that the TBS buffer, with its higher pH, stabilizes the VLPs and/or hinders maturation. TEM visualization of the sample of VLPs resuspended in TBS showed the presence of abundant particles of the size (25–30 nm diameter) expected for ACNNV VLPs (Figure 2). A yield of 10 mg/kg fresh weight of the highly purified VLPs was calculated based on the protein content of the sample.

### Expression of NNV VLPs in Tobacco BY-2 Cells

**FIGURE 3:**
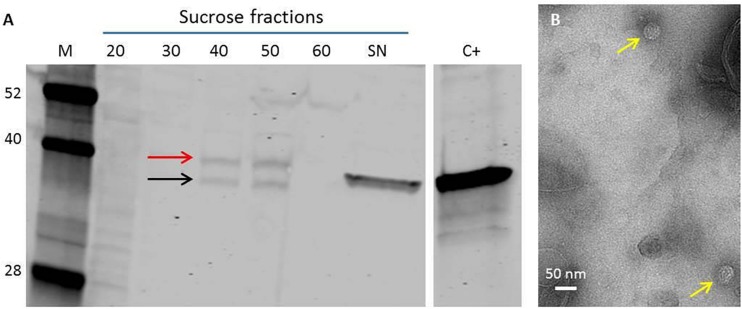
NNV VLP purification from transgenic tobacco BY-2 cell line. **(A)** The VLPs were purified through a discontinuous sucrose gradient of 20–60% (w/v), each fraction was retrieved and analyzed on a Western blot. SN = Supernatant. C+ = plant-made NNV-VLPs. Anti-NNV (ab26812) antibody. *M* = Protein ladder, molecular weights indicated. Red arrow indicates NNV coat protein alpha. Black arrow indicates mature NNV coat protein beta. **(B)** TEM image of NNV VLPs purified from transgenic BY-2 cells. Yellow arrows indicate NNV VLPs.

Since previous studies had shown that the pEAQ vectors were suitable for expression in transgenic tobacco BY-2 cells (Sun et al., 2011), we investigated the use of such cells as an alternative to expression in whole *N. benthamiana* plants. Stable transformation of tobacco BY-2 cells resulted in the production of several calli expressing the ACNNV coat protein as shown by western blot analysis using anti-NNV antibody. BY-2 cell line 16 displayed the highest concentration of NNV coat protein and thus was chosen for larger scale VLP production. To this end, a 500 ml culture of line 16 was produced and VLPs were isolated from the cells and purified through a discontinuous sucrose cushion. Western blot analysis of the sucrose gradient fractions suggested that the coat protein assembled into VLPs and there was evidence of the cleavage of the alpha to the beta protein (Figure 3A). TEM analysis of the purified material indicated that VLPs had been formed (Figure 3B) but these were only present in small amounts compared with the levels produced in whole plants.

### Cryo-EM Analysis of Plant-Produced ACNNV VLPs

**FIGURE 4:**
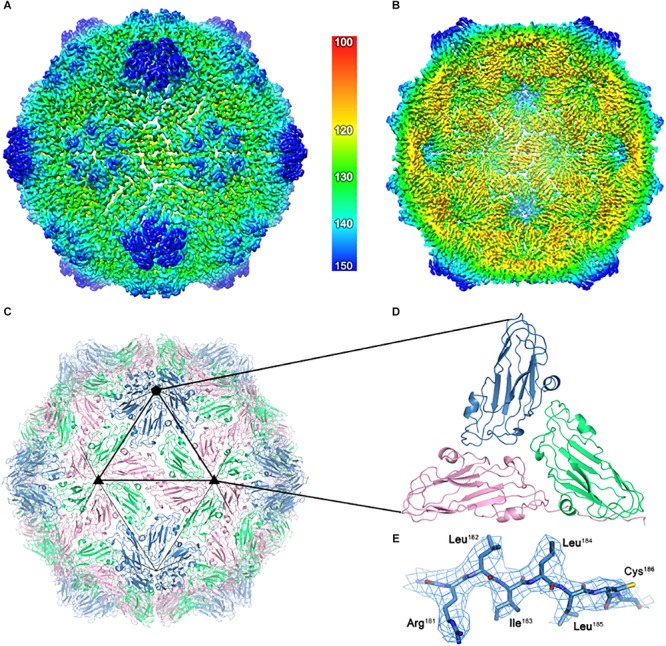
The cryo-EM structure of the ACNNV capsid. **(A)** An isosurface representation (3σ) of the 3.7 Å ACNNV-LP structure viewed down an icosahedral twofold axis with the internal view shown in panel **(B)**. The radial coloring scheme is shown in angstroms. **(C)** The atomic model of the shell component of the ACNNV *T* = 3 capsid. **(D)** Enlarged view of a single asymmetric unit showing the three quasi-equivalent conformations of the coat protein. **(E)** Representative EM density containing the refined atomic model.

**FIGURE 5:**
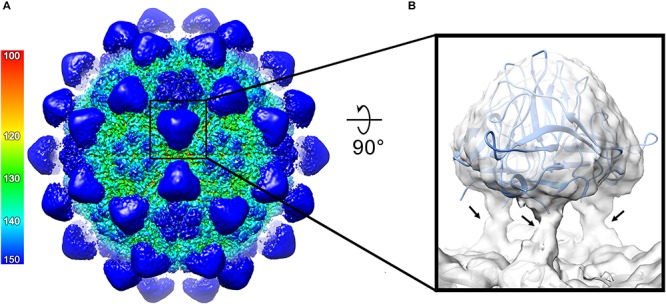
Structure of the ACNNV P-domains. **(A)** An isosurface representation (1.8σ) of the 3.7 Å ACNNV-LP structure viewed down an icosahedral two-fold axis, showing the 60 P-domain trimers. The radial coloring scheme is shown in angstroms. **(B)** Enlarged view of a single P-domain trimer from the unsharpened map containing the fitted crystal structure of the GNNV P-domain trimer. The flexible linker regions which connects the P-domains to the shell domains are indicated by arrows.

For a detailed structural analysis of the plant- produced ACNNV VLPs, cryo-EM was performed on the sample prepared from *N. benthamiana* leaves. Images of unstained, frozen-hydrated ACNNV VLPs were imaged using an electron microscope fitted with a direct electron detecting camera (Supplementary Figure S1A). Single-particle image processing was carried out in Relion, which allowed us to determine the solution structure of the VLP to a global resolution of 3.7 Å (Supplementary Figure S1B). The ACNNV VLP is an isometric particle with *T* = 3 symmetry (Figures 4A,B), consistent with previously reported structures of the grouper nervous necrosis virus (GNNV) VLPs which share ∼85 % sequence identity (Cheng et al., 1994; Chen et al., 2015). The shell domain displays the highest local resolution (Supplementary Figure S1C) which allowed the building of an atomic model of the capsid shell (Figures 4C,D). The viral asymmetric unit is composed of three quasi equivalent copies of the coat protein which have a characteristic jelly roll topology, with clear side-chain density (Figure 4E). Three P-domains of the capsid protein interact and form spikes on the particle surface (Figure 5A). The P-domains are attached to the rest of the capsid protein by a linker region (Figure 5B) and display lower local resolution (Supplementary Figure S1D), presumably as a result of flexibility. Overall, the cryo-EM analysis confirmed that the structure of the plant-produced VLPs is very similar that of material produced in other expression systems.

### Plant-Made NNV VLPs Protect Sea Bass Against Viral Challenge

To investigate the immunogenicity of the plant-produced ACNNV VLPs, sea bass (*D. labrax)* were vaccinated with purified VLP preparations. Serum recovered from mock-vaccinated control and ACNNV VLP-vaccinated fish injected by either the IP or IM route showed no statistically significant levels of specific anti-betanodavirus antibodies by ELISA (Supplementary Figure S3). Despite a lack of detectable humoral response in vaccinated fish, the challenge was undertaken based on the potential for protection as a result of cellular (i.e., antibody-dependent cell-mediated cytotoxicity) immunity and the possibility that the ELISA using heterologous capture antigen (RGNNV) was not informative for ACNNV VLP- vaccinated fish despite these genogroups sharing serogroup C.

The first clinical signs of inappetence and reduced activity were observed on the 5th day post-exposure in a small number of individuals in both tanks. This was followed a day later by loss of equilibrium and spiral swimming leading to mortality or moribundity, and removal from the experiment of a total of 5 and 11 fish from each tank, respectively, all of which were sham-vaccinated controls. Henceforth the term mortality will be used to refer to actual mortalities and humanely terminated moribund animals, the latter of which represent the majority of removed animals.

**FIGURE 6:**
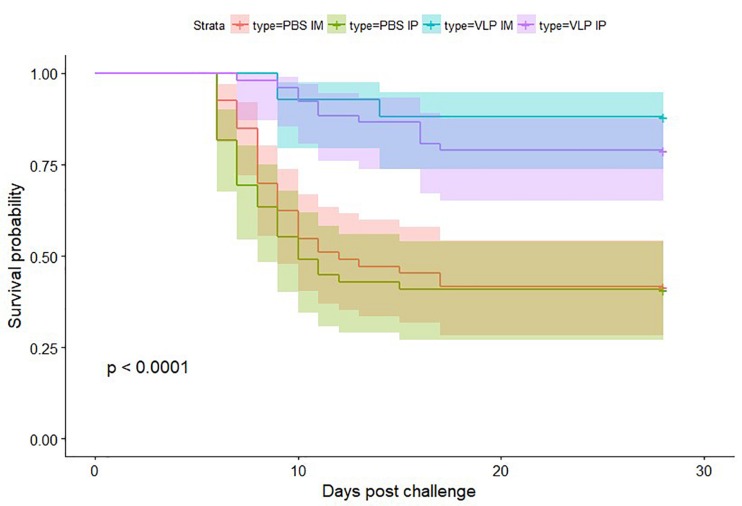
Kaplan–Meier curves showing survival probability after betanodavirus challenge. Color-coded shaded areas indicate 95% confidence intervals.

Survival probabilities by group are represented with the Kaplan–Meier survival plot (Figure 6). Pairwise comparison demonstrated no significant difference between replicate tanks (data not shown), thus survival analysis is shown in Figure 6 utilizing combined data. Mortality in the VLP vaccinated groups was significantly lower (*p* < 0.05) than in the sham vaccinated controls. Pairwise comparison showed no significant difference in vaccination route.

Cumulative mortality in the IP and IM sham vaccinated controls reached between 57.1 and 60.8% (Table 1). First challenge mortality in vaccinated fish was delayed by approximately 2 or 4 days for IP-vaccinated and IM-vaccinated fish, respectively. In contrast, cumulative mortality in the IP VLP vaccinated groups reached only 20.7 and 20.8% in the duplicate tanks, and cumulative mortality in the IM VLP vaccinated groups reached only 7.7 and 13.0% in the duplicate tanks, respectively (Table 1). This represents a relative percent survival at the end of the study [(RPSend) Amend, 1981] for vaccinated fish ranging from 63.6 to 86.5% with the IM route being the more effective (Table 1). The data indicate that the plant-made VLPs, even in the absence of immune promoting adjuvant, can significantly protect sea bass against NNV.

**TABLE 1:** Cumulative mortality (%) and relative percent survival (RPS_*end*_) for IP and IM administered VLP vaccine.

	**Cumulatve mortality (%)**		
**Tank – vaccine delivery route**	**Vaccinated**	**Mock vaccinated**	**RPS_*end*_**
T07-05 (A) – IP	20.7	60.8	65.9
T07-05 (A) – IM	7.7	57.1	86.5
T07-04 (B) – IP	20.8	57.1	63.6
T07-04 (B) – IM	13.0	60.0	78.3

## Discussion

Several vaccine candidates against NNV have been developed but none has been commercialized yet. However, the expanding world population and overfishing of the oceans has led to a growing demand for farmed fish, making it a very high value industry. NNV causes mortality rates up to 99% particularly in juvenile stages and is a significant impediment to expanding aquaculture and food security, particularly in the Mediterranean region, thus the interest in a prophylactic treatment such as a vaccine, is high (Lin et al., 2007). The aim of this study was to investigate the feasibility of making a plant-produced NNV VLP vaccine. Initial experiments involved transiently expressing ACNNV VLPs in *N. benthamiana* plants and optimizing their expression level, purification and stability. The transiently expressed NNV VLPs accumulate to relatively high levels in plants within 4-6 dpi and yields of 10 mg purified VLPs/Kg fresh weight leaf tissue could be obtained. As an alternative, the use of tobacco BY-2 cells in culture as a production system was also investigated. The results showed that it is possible to produce lines of cells expressing the ACNNV coat protein and that the protein assembled into VLPs. However, as the yield was relatively low, subsequent characterisation was carried out on the VLPs purified from *N. benthamiana* leaves.

The relatively simple situation where only one protein that self-assembles into an icosahedral structure of 180 copies makes them an attractive model for studies of VLP production in plants. The high-resolution structural studies revealed that the plant-made ACNNV VLPs are of isometric shape with *T* = 3 symmetry, consistent with previously reported structures and authentic-looking when compared to the wild-type NNV. These data confirm the previous conclusion that plants are an effective way of producing VLPs (Marsian and Lomonossoff, 2016). Protection against NNV was carried out in sea bass with a view to determining whether the plant-produced VLPs could serve as a candidate vaccine in target animals. Despite a lack of a detectable humoral response, the plant-produced VLPs, in the absence of immune stimulating adjuvant, were shown to confer moderate to strong protection in virus-challenged fish. Hodgins et al. (2017) demonstrated that a plant-expressed VLP vaccine protected mice from highly virulent influenza H1N1 despite an absent of humoral responses. The authors showed that cellular immune responses contributed to protection in the vaccinated animals, suggesting that the mechanism of protection may be antibody-dependent cell-mediated cytotoxicity. In the present study, the level of protection afforded is consistent with that observed in other experimental vaccines for NNV (Yamashita et al., 2005; Thiery et al., 2006) and established vaccines for other viral diseases limiting aquaculture (Gudding et al., 2014; Munang’andu and Evensen, 2019). This is encouraging and suggests that plant-based vaccines have a future for deployment in aquaculture, however, more investigations are needed to understand the mechanisms of protection.

The results presented here were achieved through parenteral administration of the candidate vaccine. However, when working with a large number of fish and at juvenile stages immersion or oral vaccination would have significant advantages. Possibilities of incorporating plant material harboring NNV VLPs into feed pellets and vaccine efficacy thereof should be investigated. Such an approach would be simple, inexpensive and rapid as it does not require purification of the VLPs. The use of cells, such as BY-2, in culture may be particularly suited to such an approach though it would be essential to optimize yields.

## Data Availability

The datasets generated for this study can be found in wwPDB, EMD-4899, PDB ID 6RJ0.

## Ethics Statement

All experimental animal use was performed in accordance with UK Home Office Regulations under the Animals (Scientific Procedures) Act 1986, with scrutiny and approval by the Cefas Weymouth Laboratory local Animal Welfare Ethical Review Body (AWERB).

## Author Contributions

GL, AR, RP, and NR conceived and planned the study. JM, AR, and DH performed the VLP expression and characterisation experiments, and analyzed the data. RP and IC planned and performed the experiments and sample analysis for immunization and challenge in fish. All authors contributed key ideas, analyzed the data, and wrote the manuscript.

## Conflict of Interest Statement

AR is employed by VTT, a non-profit liability company. GL declares that he is a named inventor on granted patentWO29087391 A1 which describes the transient expression system used in this manuscript to express the ACNNV VLPs.

The remaining authors declare that the research was conducted in the absence of any commercial or financial relationships that could be construed as a potential conflict of interest.

## Footnotes

1 http://www.mrc-lmb.cam.ac.uk/kzhang/

## Supplementary Material

FIGURE S1 Cryo-EM analysis of ACNNV-LPs. **(A)** Typical micrograph from the ACNNV-LP dataset (scale bar = 50 nm). **(B)** The plot of the Fourier shell coefficient (FSC). Based on the 0.143 criterion for the gold standard comparison of two independent data sets, the resolution of the reconstruction is 3.7 Å. **(C)** An isosurface representation (3σ) of the 3.7 Å ACNNV-LP structure viewed down an icosahedral two-fold axis and colored according to local resolution. **(D)** An isosurface representation (1.2σ) of the 3.7 Å ACNNV-LP structure viewed down an icosahedral twofold axis and colored according to local resolution. The local resolution coloring scheme is shown in angstroms.

FIGURE S2 Alignment of the amino acid sequences of the ACNNV and GNNV coat proteins.

FIGURE S3 ELISA results: Box and whisker plots showing log transformed average optical density (*n* = 10) of serum samples from preimmune, sham control (PBS) and nodavirus VLP vaccinated fish by IM and IP administration routes. Positive controls are known positive sera from previous challenges.

FIGURE S4 Schematic diagram showing vaccination and challenge schedule. d: days; DD: degree days; IP: intraperitoneal; IM: intramuscular.

TABLE S1 Data-collection parameters and model statistics.
