# Supplementary figures and images for: Plant-Made Nervous Necrosis Virus-Like Particles Protect Fish Against Disease
Source: Front Plant Sci. 2019 Jul 9;10:880. doi: 10.3389/fpls.2019.00880 (PMC6629939; doi:10.3389/fpls.2019.00880)

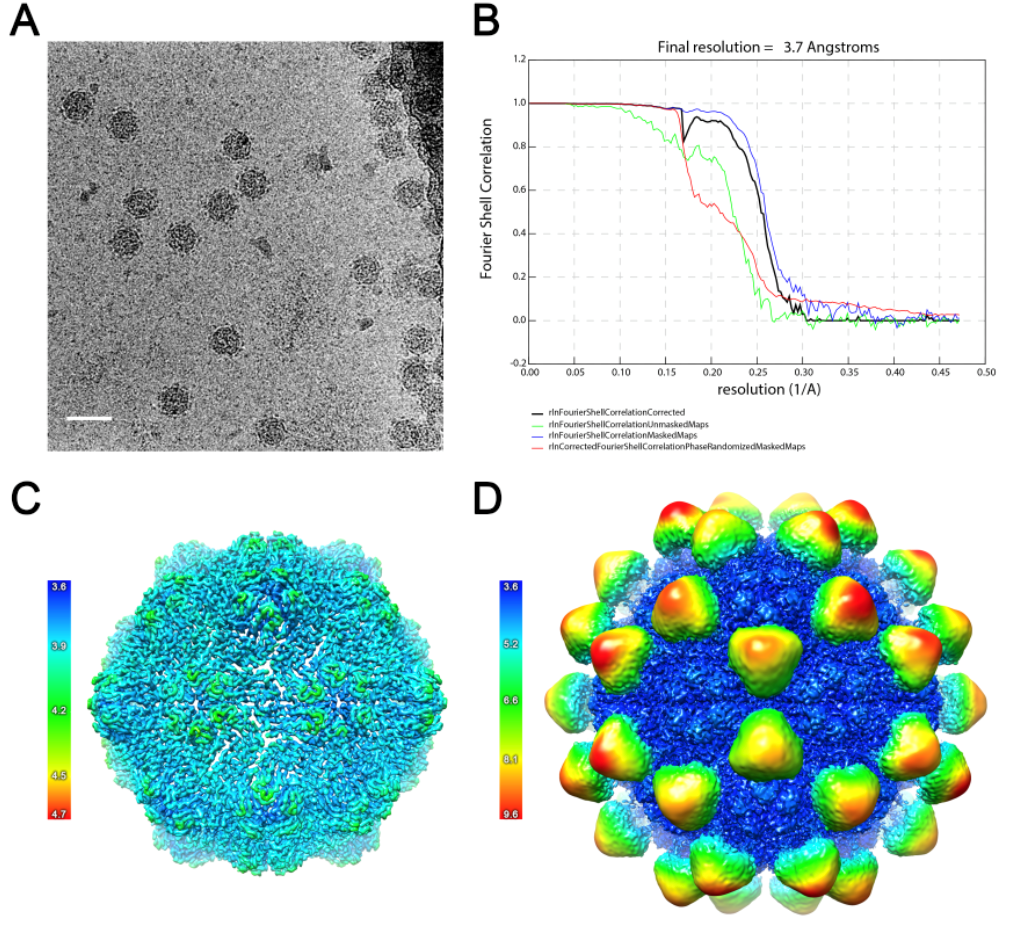

Supplement: FIGURE S1 — Cryo-EM analysis of ACNNV-LPs. (A) Typical micrograph from the ACNNV-LP dataset (scale bar = 50 nm). (B) The plot of the Fourier shell coefficient (FSC). Based on the 0.143 criterion for the gold standard comparison of two independent data sets, the resolution of the reconstruction is 3.7 Å. (C) An isosurface representation (3σ) of the 3.7 Å ACNNV-LP structure viewed down an icosahedral two-fold axis and colored according to local resolution. (D) An isosurface representation (1.2σ) of the 3.7 Å ACNNV-LP structure viewed down an icosahedral twofold axis and colored according to local resolution. The local resolution coloring scheme is shown in angstroms. [file Image_1.TIF]

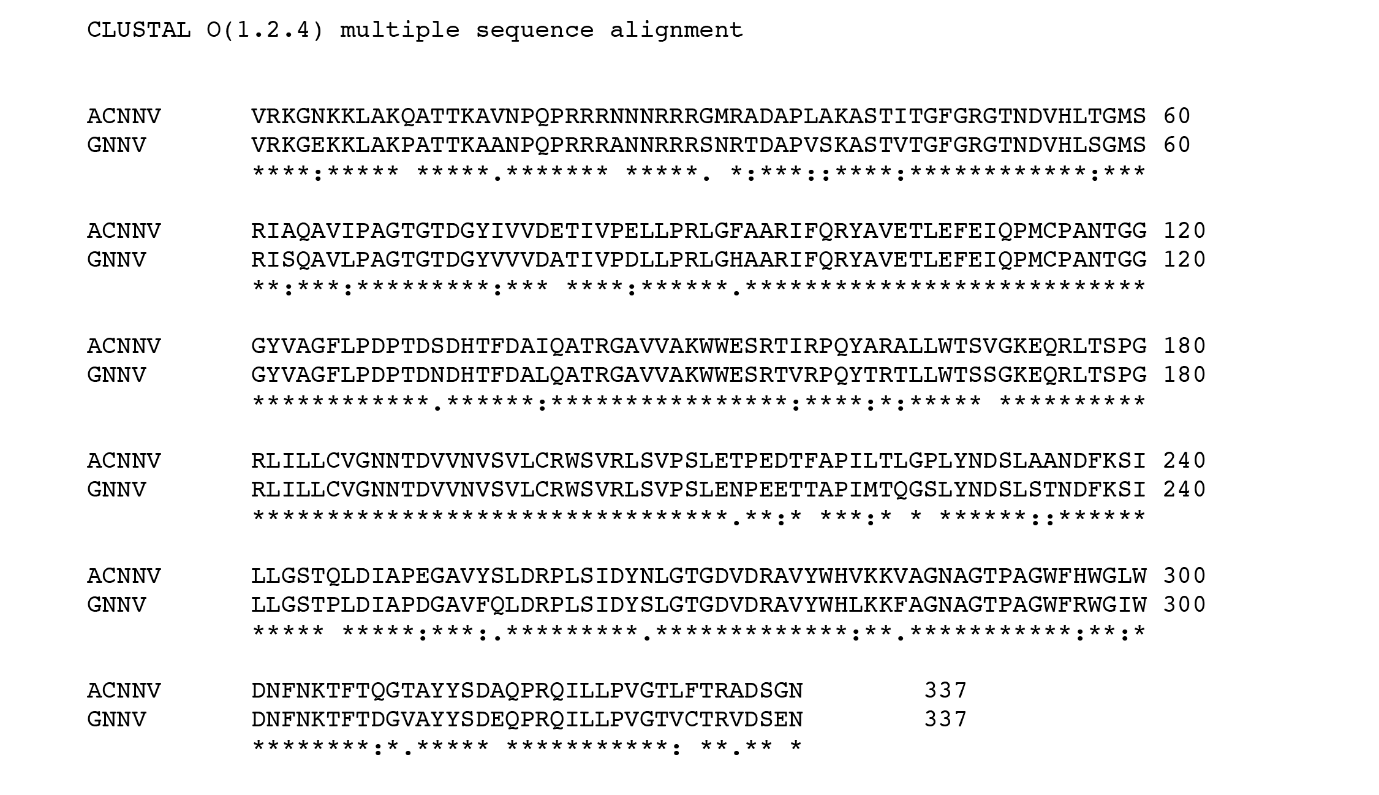

Supplement: FIGURE S2 — Alignment of the amino acid sequences of the ACNNV and GNNV coat proteins. [file Image_2.TIF]

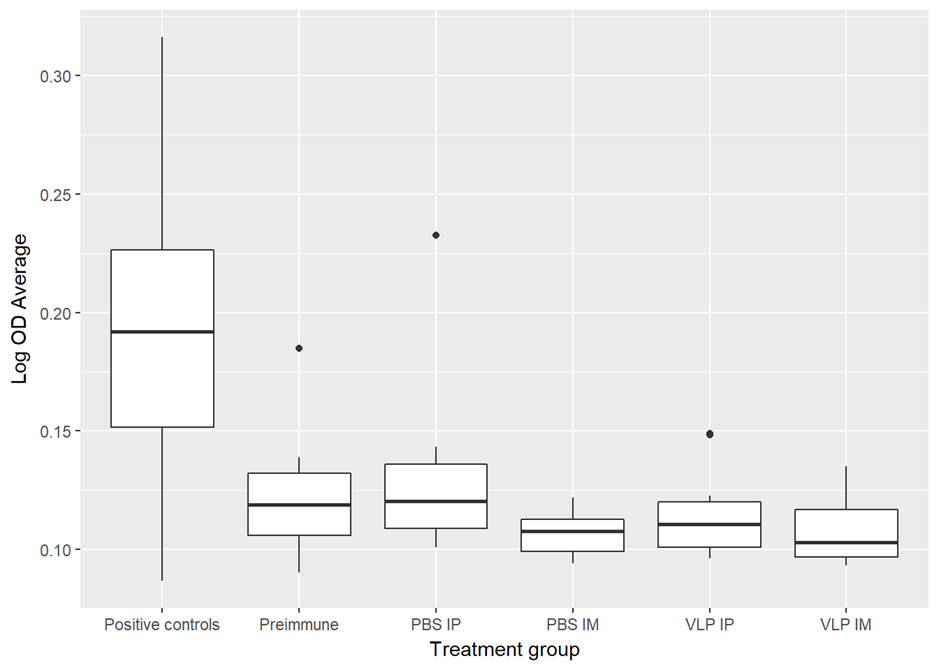

Supplement: FIGURE S3 — ELISA results: Box and whisker plots showing log transformed average optical density (n = 10) of serum samples from preimmune, sham control (PBS) and nodavirus VLP vaccinated fish by IM and IP administration routes. Positive controls are known positive sera from previous challenges. [file Image_3.TIF]

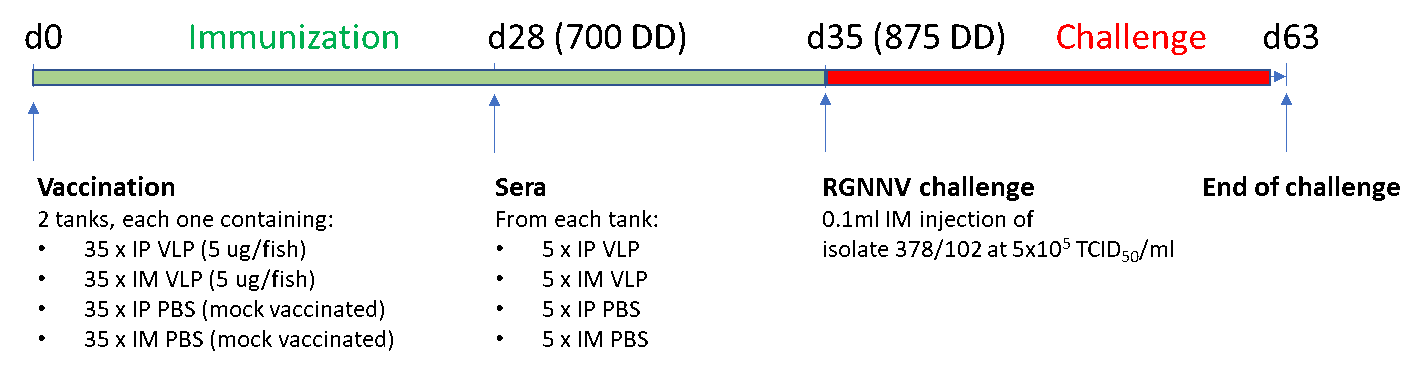

Supplement: FIGURE S4 — Schematic diagram showing vaccination and challenge schedule. d: days; DD: degree days; IP: intraperitoneal; IM: intramuscular. [file Image_4.PNG]
