# Supplementary material for: Plant-Made Nervous Necrosis Virus-Like Particles Protect Fish Against Disease
Source: Front Plant Sci. 2019 Jul 9;10:880. doi: 10.3389/fpls.2019.00880 (PMC6629939; doi:10.3389/fpls.2019.00880)
Supplement: TABLE S1 — Data-collection parameters and model statistics. [file Table_1.DOCX]

**Supplementary Table 1: Data-collection parameters and model statistics.**

| Data Collection | |
| --- | --- |
| Microscope | FEI Titan Krios |
| Camera | Gatan K2 Summit |
| Voltage | 300 keV |
| Pixel Size | 1.06 Å |
| Total Dose | 45 |
| Number of Frames | 20 |
| Defocus Range | -0.5 to -2.5 |
| Micrographs | 2359 |
| Acquisition software | FEI EPU |
| Image Processing | |
| Motion correction | MotionCor |
| CTF estimation | Gctf |
| Particles selected | 20,887 |
| Reconstruction | |
| Software | RELION 2.0 |
| Particles contributed | 16,616 |
| B-factor | -137 |
| Resolution (FSC 0.143) | 3.7 Å |
| Model Building and Refinement | |
| Model Refinement software | Phenix real space |
| Map CC (whole unit cell) | 0.69 |
| Map CC (all atoms) | 0.80 |
| R.M.S.D Bond length | 0.01 |
| R.M.S.D Bond angles | 1.07 |
| Ramachandran Preferred | 86.21 % |
| Ramachandran Allowed | 13.79 % |
| Ramachandran Outlier | 0.00 % |
| Rotamer Outliers | 0.00 % |
| C-beta deviations | 0 |
| All-atom clashscore | 3.16 |
